# Oxidative Stress in Bacteria and the Central Dogma of Molecular Biology

**DOI:** 10.3389/fmolb.2021.671037

**Published:** 2021-05-10

**Authors:** Michel Fasnacht, Norbert Polacek

**Affiliations:** ^1^ Department of Chemistry, Biochemistry and Pharmaceutical Sciences, University of Bern, Bern, Switzerland; ^2^ Graduate School for Cellular and Biomedical Sciences, University of Bern, Bern, Switzerland

**Keywords:** oxidative stress, ROS, oxidative damage, DNA damage, RNA damage, protein damage

## Abstract

Ever since the “great oxidation event,” Earth’s cellular life forms had to cope with the danger of reactive oxygen species (ROS) affecting the integrity of biomolecules and hampering cellular metabolism circuits. Consequently, increasing ROS levels in the biosphere represented growing stress levels and thus shaped the evolution of species. Whether the ROS were produced endogenously or exogenously, different systems evolved to remove the ROS and repair the damage they inflicted. If ROS outweigh the cell’s capacity to remove the threat, we speak of oxidative stress. The injuries through oxidative stress in cells are diverse. This article reviews the damage oxidative stress imposes on the different steps of the central dogma of molecular biology in bacteria, focusing in particular on the RNA machines involved in transcription and translation.

## Introduction

### Sources of Oxidative Stress and the Bacterial Defense Mechanisms Against Them

The earliest single-celled life forms evolved on Earth in an anoxic environment around 4 billion years ago (Orgel, 1998). It was only after the “great oxidation event” approximately 2.4 billion years ago that, due to the emergence of photosynthesis, the oxygen level of Earth’s atmosphere rose significantly (Lyons et al., 2014). Consequently, cellular life forms had to adapt to the potential danger of harmful by-products of oxygen metabolism, better known as reactive oxygen species (ROS). ROS can damage all types of cellular components like nucleic acids, proteins and lipids (Imlay, 2013). Two of the most widely studied forms of ROS, superoxide (O_2_^−^) and hydrogen peroxide (H_2_O_2_), are constantly produced endogenously through the autoxidation of O_2_ on a range of both aerobic and non-aerobic respiratory flavoproteins (Imlay, 1995; Messner and Imlay, 1999; Kussmaul and Hirst, 2006; Korshunov and Imlay, 2010), as well as on non-respiratory flavoproteins (Massey et al., 1969; Geary and Meister, 1977; Grinblat et al., 1991). To protect the cells against these harmful compounds, bacteria evolved enzymes termed superoxide dismutases (SODs) to convert O_2_^−^ into O_2_ and H_2_O_2_, as well as catalases and peroxidases to remove H_2_O_2_ to continuously neutralize the endogenously produced ROS (Imlay, 2008). In fully aerated *Escherichia coli*, the interplay between endogenous production and scavenging enzymes results in a steady-state intracellular concentration of ∼0.2 nM O_2_^−^ and ∼50 nM H_2_O_2_ (Imlay, 2013). Internal concentrations can however be increased by exogenous sources. The membrane of bacteria is semipermeable for H_2_O_2_. Naturally produced H_2_O_2_ can therefore enter and potentially damage bacterial cells. Sources of H_2_O_2_ can be H_2_O_2_ generated through photochemistry in surface waters (Wilson et al., 2000b; Wilson et al., 2000a) (including in common lab media (Li and Imlay, 2018)), oxic-anoxic interfaces (e.g., near the intestinal epithelium) (Imlay, 2019), excretion from lactic acid bacteria (Tong et al., 2007; Liu et al., 2011; Bao et al., 2017), and potentially indirectly from phagocytes, which produce superoxide through an NADPH oxidase (Segal, 2008). O_2_^−^ itself is not permeable through cytosolic membranes at neutral pH and can therefore not penetrate the bacterial cell enclosed in the phagosomes. One hypothesis is that the O_2_^−^ dismutates spontaneously to H_2_O_2_ to exert the observed toxic effect on the captured bacteria in the phagocyte (Imlay, 2019). Due to the membranes’ impermeability to superoxide, other methods than simple diffusion must be used both in the lab and in nature to increase intracellular O_2_^−^ concentration in bacteria. Redox-cycling antibiotics, such as the synthetic viologen paraquat or the naturally occurring phenazines and quinones, are able to penetrate bacterial cells. Inside the cell, they oxidize redox enzymes and produce O_2_^−^ by transferring the electrons to oxygen (Turner and Messenger, 1986; Kuo et al., 1987; De Paiva et al., 2003; Inbaraj and Chignell, 2004). A unique source of ROS is found in phototrophic organisms. During photosynthesis, both O_2_^−^ and H_2_O_2_ are produced at the photosystem I (Asada, 1999). Additionally, singlet oxygen (^1^O_2_) is generated at the photosystem II by a transfer of light energy to oxygen (Zolla and Rinalducci, 2002; Nishiyama et al., 2006; Glaeser et al., 2011). Singlet oxygen, another relevant ROS species, has a short half-life and diffuses freely through cells. Measurements of singlet oxygen travel distances in its lifetime determined that in microorganisms, ^1^O_2_ can theoretically be distributed throughout the whole bacterial cell once it is produced in the photosystems of the cytosolic membrane (Skovsen et al., 2005). In cyanobacteria, photosystems I and II are assisted by light-harvesting, antenna-like protein complexes called phycobilisomes (Singh et al., 2015). Close investigation of the ROS generated at phycobilisomes isolated from *Synechocystis* sp. PCC 6803 revealed that both a Type 1 and Type 2 photochemistry reaction (Foote, 1991) took place, resulting in O_2_^−^ and ^1^O_2_ respectively (Rinalducci et al., 2008). While this list of ROS sources does not comprise the whole universe of potentially harmful ROS for bacteria (e.g., lipid peroxidation (Wang et al., 2004; Wang et al., 2006; Hong et al., 2019), ozone (Patil et al., 2011; Li et al., 2020)), this review focuses on the most widely studied forms of ROS to summarize our current understanding of oxidative stress damage and response in bacteria.

Oxidative stress can result in damage of both the backbone and bases of nucleic acids, both free and incorporated oxidized amino acids, as well as cofactors of proteins. To mitigate the damage of oxidative stress on cell biology, different stress response regulons are activated in bacteria, depending on the type of stressor (reviewed in (Imlay, 2015)). OxyR, a transcription factor widely distributed among gram-negative bacteria, is directly induced by H_2_O_2_. In most cases, the activated form of OxyR recruits RNA polymerase to transcribe about thirty different stress response genes (Seo et al., 2015), but there are also cases found in some bacteria where OxyR acts as a repressor to prevent the transcription of these genes under unstressed conditions (Loprasert et al., 2000; Luo et al., 2005; Chen et al., 2008; Ieva et al., 2008; Heo et al., 2010; Teramoto et al., 2013). PerR is an alternative transcription factor to OxyR, which is often found in gram-positive bacteria such as *Bacillus subtilis* (Jacquamet et al., 2009), but reports on PerR homologues in gram-negative bacteria, such as *Campylobacter jejuni* and *Synechocystis* sp. strain PCC 6803, have been published as well (Van Vliet et al., 1999; Li et al., 2004). The regulon of PerR contains most of the same stress response genes as the OxyR regulon (Helmann et al., 2003). Both induce enzymes to scavenge H_2_O_2_ and therefore mitigate further oxidative damage. However, only the OxyR regulon includes disulfide-reducing redoxins to repair already damaged proteins (Imlay, 2015; Seo et al., 2015). Two successive transcription factors are employed by *E. coli* to defend cells against the threat of increased O_2_^−^ concentrations (Blanchard et al., 2007). SoxR is first activated by rising superoxide levels and induces the transcription of SoxS. SoxS in turn promotes the transcription of a defensive regulon encompassing 25 proteins to prevent the entry and accumulation of redox-active molecules in the cell (Blanchard et al., 2007; Seo et al., 2015). The SoxRS organization as found in *E. coli* is not a universal trait of all bacteria. In fact, performing a BLAST search for both SoxR and SoxS homologues in the bacterial domain revealed that SoxR is restricted to Proteobacteria and Actinobacteria, while SoxS was exclusively found in the family Enterobacteriaceae (Dietrich et al., 2008). The model bacterial system for the response to photooxidative stress through ^1^O_2_ is the anoxygenic anaerobic photosynthetic *Rhodobacter sphaeroides*. In *R. sphaeroides*, the ^1^O_2_ response regulon is activated through a cascade of transcription factors. First, the alternative sigma factor RpoE gets activated. While the RpoE regulon itself is rather small (Glaeser et al., 2007), it includes two additional sigma factors, RpoH_I_ and RpoH_II_, which in turn activate the stress response regulon against singlet oxygen (Nuss et al., 2009; Nuss et al., 2010).

**FIGURE 1:**
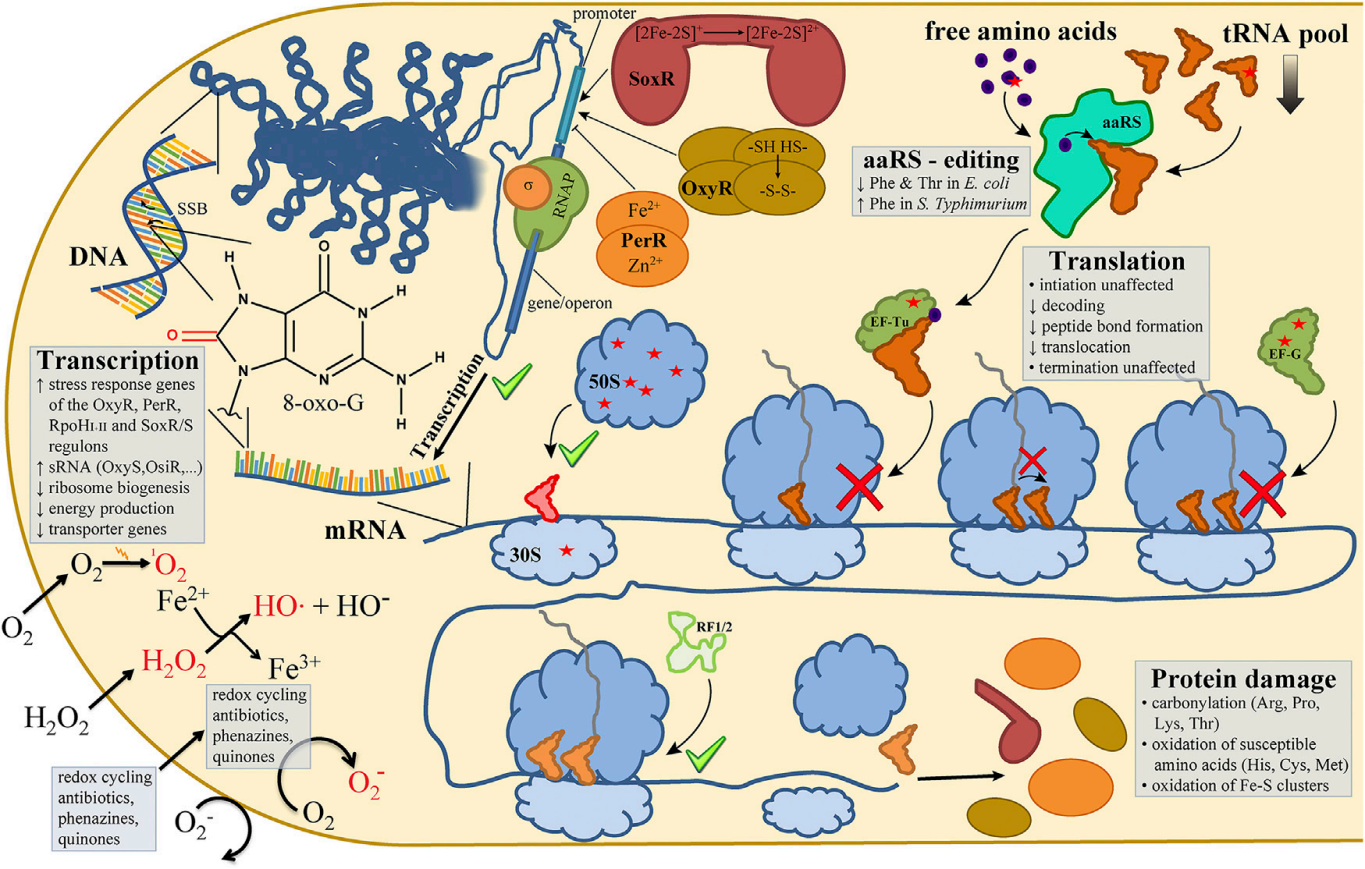
Oxidative stress in bacteria affects every sub step of the central dogma. In bacterial cells the most widely studied reactive oxygen species (ROS) are ^1^O_2_ (singlet oxygen), O_2_^−^ (superoxide) and H_2_O_2_ (hydrogen peroxide), with the latter giving rise to HO• (hydroxyl radicals) via the Fenton reaction **(lower left)**. ROS-dependent oxidations of DNA nucleobases (e.g., 8-oxo-G) or DNA backbone lesions (single-strand breaks; SSB) are recognized and trigger transcriptional responses mediated by oxidative stress-induced transcription factors (e.g., OxyR, PerR, SoxR) **(upper left)**. Bacterial translation is affected in multiple ways by oxidative lesions (red asterisks) as observed in the pool of free amino acids, in elongation factors (EF-Tu, EF-G), ribosomal proteins, and in the main RNA species of the translation machinery (tRNA, rRNA) **(upper right and center of the figure)**. Not necessarily expected, the sub steps of the ribosomal elongation cycle of protein biosynthesis are not affected by ROS to the same extent (center and lower right). While translation initiation and termination do not seem to be markedly inhibited (green check marks), A-site tRNA accommodation, peptide bond formation and EF-G-mediated tRNA translocation are inhibited by oxidative lesions (red cross signs). Please refer to the main text for more details on the ROS-mediated effects on the sub steps of the central dogma.

These defensive systems can be highly effective, allowing an exponentially growing *E. coli* culture to survive and overcome an extracellular H_2_O_2_ concentration that is 10^6^ times the normal intracellular H_2_O_2_ concentration produced by endogenous activity (Zhu and Dai, 2019). Interestingly, cyanobacteria, which initially caused the great oxidation event (Schirrmeister et al., 2015), are much more susceptible to H_2_O_2_ as compared to eukaryotic aquatic microorganisms (Drábková et al., 2007; Mikula et al., 2012; Leunert et al., 2014; Liu et al., 2017). This higher susceptibility to H_2_O_2_ might potentially be exploited to combat increased cyanobacteria blooms caused by global warming (Chen et al., 2021). Notwithstanding the effectiveness of the response system, damage through oxidative stress can be acquired extremely quickly and acts non-discriminatory on all cellular compounds. In the next chapters, we will review how exactly oxidative damage influences different classes of biomolecules. In particular, we will focus on the damage that oxidative stress inflicts on the different steps of the extended central dogma of molecular biology in bacteria, which describes the flow of genetic information in a cell (Figure 1).

## Genetic Information is Stored in DNA

DNA cannot be damaged directly neither by H_2_O_2_ nor by O_2_^−^ alone (Winterbourn, 2013). Nevertheless, mutants lacking SODs (Farr et al., 1986) or catalases and peroxidases (Park et al., 2005) show increased mutagenesis rates. The mutagenic effect of H_2_O_2_ is easily explained by the Fenton reaction (Fenton, 1894) mediated by Fe^2+^-ions associated with the DNA:

$${\text{Fe}}^{2+}+{\text{H}}_{2}{\text{O}}_{2}\to {\text{Fe}}^{3+}+{\text{HO}}^{-}+\text{HO}\cdot$$

The resulting hydroxyl radical is short-lived due to its high reactivity. It can react with most biomolecules at almost diffusion-limited rates. When produced in close proximity to DNA, hydroxyl radicals can damage both the nucleobase or the deoxyribose moieties, leading to mutations and strand breaks (Hutchinson, 1985; Dizdaroglu et al., 1991; Evans et al., 2004). Fittingly, Rai *et al.* found Fe^2+^ preferably associated to specific short DNA sequences (Rai et al., 2001) that had previously been found to be preferentially cleaved under oxidative stress conditions (Henle et al., 1999). A commonly used marker for oxidative stress damage is 8-oxo-7,8-dihydroguanosine (8-oxo-G), the most commonly observed oxidation product of a guanine base (Figure 1). Guanine has a lower reduction potential than the other DNA bases. Nearby oxidized bases can therefore readily be repaired by an electron transfer from the guanine base to the oxidized base (Candeias and Steenken, 1993), effectively transferring the oxidation site onto the guanine. 8-oxo-G can base-pair with both a cytosine or adenine base, thus affecting the coding potential of DNA (Cheng et al., 1992). Crystal structures of a *Bacillus stearothermophilus* DNA polymerase with 8-oxo-G pairing to both the cognate and mismatched base showed that under these conditions the proofreading mechanisms of the DNA polymerase are no longer effective (Hsu et al., 2004). In fact, Hsu *et al.* observed an inversion of the ordinary mismatch recognition. The in theory canonical 8-oxo-G:cytosine base-pair behaved as a mismatch, whereas the 8-oxo-G:adenine base-pair was recognized as cognate, which ultimately leads to a G to T transversion in the replicated DNA strand.

The impact of O_2_^−^ on DNA is less obvious. In the end, the observed DNA damage is again due to the Fenton reaction described above. The involvement of superoxide is however only indirect. Keyer *et al.* showed that SOD deficient bacterial strains had increased free intracellular iron concentrations (Keyer and Imlay, 1996). The authors were also able to show that O_2_^−^ excised the iron from a family of dehydratases that contain [4Fe-4S] clusters. Additionally, O_2_^−^ was shown to reduce Fe^3+^ to Fe^2+^, regenerating ferrous iron for a continuous Fenton reaction (Fong et al., 1976). Finally, O_2_^−^ is converted to H_2_O_2_ through the actions of SODs. The increase in both Fenton reaction educts results in the observed higher mutagenesis rate. Taken together, these findings highlight the need of bacterial cells to properly regulate their intracellular pool of free iron as well as iron associated and bound to macromolecular complexes. Unsurprisingly, both the transcription factors OxyR and PerR induce the transcription of an iron import repressor (*fur*) and a DNA-binding mini-ferritin (*dps*, *mrgA* in *B. subtilis*) to minimize free iron under conditions of oxidative stress (Imlay, 2015). The SoxRS system also activates Fur but has no effect on Dps. Instead it stimulates transcription of the endonuclease IV (*nfo*) gene for DNA repair (Seo et al., 2015). Similarly, iron-binding proteins are upregulated through the RpoE/RpoH_I/II_ sigma factors cascade in *R. sphaeroides*, while uptake mechanisms for Fe^2+^ are minimized (Glaeser et al., 2007; Nuss et al., 2009).

In contrast to O_2_^−^, singlet oxygen can directly react with DNA. Unlike HO∙, which targets all nucleobases, ^1^O_2_ exclusively interacts with guanine, which contains conjugated, electron-rich double bonds (Ravanat et al., 2001). The resulting oxidation product of the base is however again 8-oxo-G (Cadet et al., 2006; Cadet et al., 2010).

## DNA is Transcribed to RNA

In bacteria, the genetic information stored in DNA is transcribed to RNA by a single RNA polymerase (RNAP) structurally similar to eukaryotic RNA polymerase II (Ebright, 2000). Specificity of gene expression is modulated by the use of different sigma (σ) factors and an array of transcription factors such as the above mentioned OxyR, PerR, and SoxR/S (Figure 1). The fact that the stress response genes in their regulons are actively transcribed and translated as a response to oxidative stress is evidence that the RNAP is not incapacitated under these conditions. The aforementioned transcription factors themselves are activated by oxidation. For example, H_2_O_2_ oxidation of OxyR induces the formation of an intramolecular disulfide bond, which results in a significant conformational change of OxyR into the DNA binding form (Choi et al., 2001). PerR, which is often found in gram-positive bacteria, generally works as a repressor. Upon oxidation, it loses the ability to bind DNA and thus enables the stress regulons to be transcribed. PerR subunits have two metal ion binding sites; the structural site which irreversibly binds Zn^2+^ and the regulatory site which reversibly binds Fe^2+^ (Herbig and Helmann, 2001). PerR is deactivated by H_2_O_2_ through metal-catalyzed oxidation of histidine residues that coordinate the Fe^2+^ ion in the regulatory site, resulting in the dissociation of PerR from DNA (Lee and Helmann, 2006; Traoré et al., 2009). The homodimer SoxR, which is first triggered in the SoxRS cascade, is activated through the oxidation of [2Fe-2S]^+^ clusters when cells are exposed to redox-cycling compounds (Gaudu et al., 1997). Whether it is O_2_^−^ that directly induces SoxR oxidation is however disputed (Imlay, 2015). Activation of the alternative transcription factor RpoE in *R. sphaeroides* by singlet oxygen is sensed by ChR, an anti-transcription factor in complex with RpoE (Campbell et al., 2007). ChR is targeted by ^1^O_2_, which results in the disassembly of the complex, releasing active RpoE (Campbell et al., 2007).

Additional evidence for the functionality of RNAP under oxidative stress is the transcription of small regulatory noncoding RNAs (sRNAs). One example found in *E. coli* is OxyS, which is induced by OxyR. This 109 nucleotides (nt) long RNA has been implicated in the regulation of as many as 40 genes and deletion of the *oxyS* locus results in higher mutagenesis rates compared to wildtype cells when treated with H_2_O_2_ (Altuvia et al., 1997). Barshishat *et al.* showed that OxyS induces a temporary cell cycle arrest, therefore allowing for proper DNA damage repair (Barshishat et al., 2018). The cell cycle arrest is prompted through the repression of the *nusG* gene upon binding of up to two OxyS molecules around the ribosome binding site on the *nusG* mRNA. NusG is a cofactor of Rho-dependent transcription and has been implicated in the suppression of prophages and other horizontally acquired portions of the genome (Li et al., 1993; Cardinale et al., 2008). The downregulation of NusG results in elevated expression of the *rac* prophage which encodes for the *kilR* gene. KilR prevents proper assembly of FtsZ, a ring-like structured protein essential for cell division (Bi and Lutkenhaus, 1991), contributing to the observed cell cycle arrest upon oxidative stress (Barshishat et al., 2018). Several additional sRNAs have been found to be induced by both ^1^O_2_ and O_2_^−^ in *R. sphaeroides* (Berghoff et al., 2009). A subset of these sRNAs were shown to be directly regulated by the RpoE/RpoH_I/II_ sigma factors cascade as a response to photooxidative stress (Berghoff et al., 2009; Nuss et al., 2010). Later reports confirmed the roles of these sRNAs in oxidative stress resistance (Adnan et al., 2015; Billenkamp et al., 2015; Peng et al., 2016).

An inverted role was described for the sRNA IsrR in the cyanobacterium *Synechocystis* sp. PCC 6803. IsrR was found to regulate the expression of the iron deficiency-stress induced protein A (IsiA), which was postulated to shade the photosystem II for oxidative stress prevention (Yeremenko et al., 2004). However, in this case, IsrR is not induced by oxidative stress but is constitutively transcribed, resulting in diminished *isiA* mRNA levels. Upon oxidative stress, IsrR levels are reduced, allowing for *isiA* mRNA accumulation and translation (Dühring et al., 2006).

## The Transcribed mRNA is Translated to Proteins

Upon a short H_2_O_2_ burst, Liu *et al.* found that in *E. coli* 8-oxo-G levels were higher in RNA than in DNA, meaning that oxidative stress has a stronger effect on RNA (Liu et al., 2012). Additionally, they showed that highly structured RNA like tRNAs (transfer RNA) and rRNAs (ribosomal RNA) are not protected from oxidative damage. This is in line with findings of other groups that oxidative stress generally inhibits the translation process (Zhong et al., 2015; Zhu and Dai, 2019). This chapter will now discuss in more detail how the different cogwheels of the translation machinery are affected by oxidative stress (Figure 1).

### Messenger RNA (mRNA)

mRNAs carry the information for the synthesis of a protein. Equal to its mutagenic effect in DNA replication, 8-oxo-G can affect the decoding process during translation. Using an *E. coli in vitro* translation system, Simms *et al.* showed that an oxidized base in the A-site codon can lead to the incorporation of a non-cognate amino acid (Simms et al., 2014). 8-oxo-G preferably adopts the *syn* conformation of the glycosidic bond in the ribosomal A-site, resulting in an 8-oxo-G:A base pair during the decoding step (Thomas et al., 2019). However, regardless of whether the incoming tRNA had a C or an A base pairing with the 8-oxo-G, translation rates at these codons in the *in vitro* system were decreased to an extent that suggests strong translational stalling *in vivo* instead (Simms et al., 2014). Incorporation of a non-cognate amino acid could lead to misfolding and protein aggregation (Drummond and Wilke, 2008). In agreement with the conclusion of Simms *et al.,* a later study found no increase in protein aggregation under oxidative stress conditions (Zhong et al., 2015). Simms *et al.* additionally found that oxidation at the wobble position, where usually non-cognate base pairing is tolerated more freely, equally affected peptide bond formation rates as oxidation at the other two positions of the codon did. Summarizing their findings, Simms *et al.* concluded that oxidations of mRNA bases inhibit the decoding step by preventing the small subunit from adopting the active conformation required for proper tRNA selection regardless of the position of the oxidative lesion in the codon. This observation, however, seems to be exclusive to mRNA-tRNA interactions in the decoding center, since peptide release mediated by bacterial release factors (RF1 and RF2) was not affected by oxidized stop codons (Simms et al., 2014). The hypothesis that mostly the elongation step of translation seems to be affected by oxidative stress is supported by findings of other groups. Zhong *et al.* found an increase of polysomes under oxidative stress conditions (Zhong et al., 2015), an observation that can be explained by uninhibited translation initiation and stalling ribosomes on the mRNA. Similar results were found in a cyanobacterium (Nishiyama et al., 2001; Nishiyama et al., 2004). In a more recent publication, Zhu *et al.* quantified the elongation rate in *E. coli* upon addition of H_2_O_2_ (Zhu and Dai, 2019). They found an immediate and dose-dependent decline of the elongation rate upon stress induction (down to one amino acid per second at 5 mM H_2_O_2_), followed by a gradual recovery to a normal elongation rate of approximately 16 amino acids per second. Furthermore, the immediate drop in elongation rate and the H_2_O_2_ dose-dependent gradual incline of the rate to normal levels correlated well with an observed dose-dependent growth arrest phase.

Generally, stress response mechanisms and changes in growth rate can have diverse effects on mRNA stability in bacteria in both global and gene-specific ways (Vargas-Blanco and Shell, 2020). To our knowledge, there is no numerical data available on mRNA degradation rates in bacteria under oxidative stress conditions. Using a DNA microarray analysis of H_2_O_2_ stressed *E. coli* cells, Zheng *et al.* reported an upregulation of the OxyR regulon and other stress response genes, as well as a downregulation of many ribosomal protein genes, cold shock genes, ATP synthase genes, and transporter genes (Zheng et al., 2001). There are however some reports on sRNAs regulating mRNA stability upon oxidative stress conditions. As discussed above, sRNAs can positively affect oxidative stress resistance (Dühring et al., 2006; Adnan et al., 2015; Billenkamp et al., 2015; Peng et al., 2016; Barshishat et al., 2018). While in the case of IsrR the degradation of the sRNA was shown to stabilize *isiA* mRNA (Dühring et al., 2006), a different mechanism was described for the sRNA OsiR found in *Deinococcus radiodurans*. OsiR also positively regulates oxidative stress tolerance, but its transcription is increased upon oxidative stress (Gao et al., 2020). OsiR targets the mRNA of KatE2, one of two KatE-type catalases found in *D. radiodurans* (Jeong et al., 2016). Under oxidative stress conditions, OsiR base-pairs with the *katE* mRNA, which results in increased mRNA transcript levels and increased translation of KatE (Gao et al., 2020).

RNA modifications have the potential to regulate mRNA stability. For example, N^6^-methyladenosine (m^6^A) can mark mRNAs for degradation in humans (Wang et al., 2014). Whereas m^6^A has been found to be an abundant mRNA modification in gram-negative bacteria, no change in the m^6^A/A ratio was observed under oxidative stress conditions in both *E. coli* and *P. aeruginosa* (Deng et al., 2015). Adenosine-to-inosine (A-to-I) RNA editing is another form of post-transcriptional RNA modification found in all domains of life (Torres et al., 2014). A recent report linked a specific A-to-I editing event on the mRNA of the flagellar filament protein *fliC* to an increased virulence in the bacterial plant pathogen *Xanthomonas oryzae pv. oryzicola* (*Xoc*) (Nie et al., 2020). Plants often generate ROS as a defensive reaction to either directly kill invading pathogens or, more importantly, work as signaling molecules for gene expression activation in the context of pathogen defense (Torres et al., 2006; Paiva and Bozza, 2014). The authors identified a serine to proline mutation (S128P) in FliC after exposing the bacteria to H_2_O_2_. The S128P editing event can potentially change filament structure and enhance biofilm formation, leading to an increased oxidative stress tolerance (Nie et al., 2020).

### Transfer RNA (tRNA)

Aminoacylated tRNAs read the different codons on mRNAs with their anticodon to deliver the correct amino acid to the ribosome. Interestingly, Liu *et al.* found that tRNAs folded into their native structure displayed even slightly higher 8-oxo-G levels than denatured tRNAs when treated with H_2_O_2_ *in vitro* (Liu et al., 2012). tRNAs are next to rRNA the second most abundant class of RNA and generally considered stable transcripts (Li et al., 2002). However, recent reports indicate that tRNA stability can be swiftly altered as a response to certain stress conditions and general demand (Svenningsen et al., 2017) In accordance, both Zhong *et al.* and Zhu *et al.* found a global reduction in full-length tRNA availability after the induction of oxidative stress by H_2_O_2_ in *E. coli* (Zhong et al., 2015; Zhu and Dai, 2019). The H_2_O_2_ dose-dependent reduction in full-length tRNAs could at least partially explain the strongly reduced elongation rate during oxidative stress conditions. Further evidence for this hypothesis is given by the observation that an overexpression of RNase D, resulting in an already initially reduced tRNA pool, lead to a hypersensitivity to H_2_O_2_. The opposite was true, when the tRNA pool was enriched by addition of a plasmid carrying extra copies of rare tRNA genes, cells became more resistant to oxidative stress (Zhu and Dai, 2019). Surprisingly, Leiva *et al.* published somewhat contradictory results to the two previous reports (Leiva et al., 2020). Instead of a global reduction of the tRNA pool, the authors found that from ten tested tRNA species only one specific tRNA (tRNA^Gly^) was downregulated under both H_2_O_2_ and paraquat stress. One explanation for this discrepancy might be the fact that all three publications used different *E. coli* strains in their studies, hinting to the possibility that the response to oxidative stress could even be strain specific. Leiva *et al.* also investigated whether the tRNA modification pattern was altered in response to the oxidative stress. They found no change at all known modification sites for the tRNA^Gly^ isoacceptors before and after addition of the stress (Leiva et al., 2020). However, the authors also pointed out that abasic sites and unknown oxidation products of modified tRNA^Gly^ nucleotides might have escaped their analysis. Another study, using UV-induced oxidative stress, did describe degradation of many post-transcriptional modifications under oxidative stress conditions (Sun et al., 2018).

### Aminoacyl-tRNA Synthetases (aaRS)

tRNAs are acylated with the correct amino acids by aaRSs. To ensure the cognate amino acid is loaded onto the 3′-CCA tail of the tRNA according to the genetic code, aaRS have evolved pretransfer and posttransfer proofreading functions (reviewed in (Ling et al., 2009)). While the aminoacylation active site of aaRSs exclude most non-cognate amino acids (pretransfer), especially structurally similar amino acids can escape this first proofreading sieve and be mistakenly added to a non-cognate tRNA. The posttransfer editing sites can correct these erroneous loadings. Oxidative stress has been shown to affect both the free amino acids (reviewed in (Stadtman and Levine, 2003)) and the editing sites of aaRSs. For example, the aromatic amino acid phenylalanine (Phe) is a prime target for oxidation through ROS. One of the potential oxidation products of Phe, meta-tyrosine (m-Tyr), has been shown to be incorporated into *E. coli* proteins at Phe codon positions if the editing site of PheRS was mutated. Additionally, the editing deficient PheRS *E. coli* strain was shown to be more sensitive to oxidative stress (Bullwinkle et al., 2014). Contradicting these findings, in *Salmonella enterica* serovar Typhimurium (*S.Typhimurium*) the PheRS displays increased proofreading activity of m-Tyr-tRNA^Phe^ under oxidative stress conditions (Steiner et al., 2019). *Salmonella* have been shown to be able to survive inside of ROS producing macrophages (Steele-Mortimer, 2008). A restructuring of the *S. Typhimurium* PheRS under oxidative stress conditions to a hyperaccurate editing version can at least partially explain the observed resilience of *Salmonella* against human ROS-mediated immune defense mechanisms.

In the case of threonyl-tRNA synthetase (ThrRS), which is prone to incorporate the near-cognate serine (Ser), the editing site contains an essential cysteine residue (Dock-Bregeon et al., 2004). Surface cysteine can readily be oxidized by ROS. Consistently, oxidative stress has been found to deactivate the editing site of ThrRS and lead to the misincorporation of Ser at Thr codons (Ling and Söll, 2010; Wu et al., 2014). If protein mistranslation due to loading of non-cognate amino acids on tRNAs under oxidative stress happens *in vivo*, increased protein aggregates due to misfolded proteins might be expected. As mentioned above, no such aggregates were detected in a later study (Zhong et al., 2015). One explanation could be that the misfolded proteins are readily captured and degraded by proteases before they aggregate. Indeed, a protease-deficient *E. coli* strain was shown to be more susceptible to H_2_O_2_. An effect that was exaggerated when Ser was added in excess (Ling and Söll, 2010). These mistranslation events and the resulting need for chaperones show why genes encoding the HSP20 chaperones have been found to be induced by H_2_O_2_ in an OxyR independent manner (Zheng et al., 2001).

### Ribosome

At the ribosome, proteins are formed by linking together the amino acids brought to the ribosome by tRNAs according to the codons of the mRNA present in the ribosomal decoding center. Bacterial ribosomes are enormous heterologous complexes consisting of a large (50S) and a small (30S) subunit. Both subunits contain RNA and protein components. Even though the ribosomal proteins outnumber the rRNA molecules (54 proteins to 3 rRNA molecules in *E. coli*), the rRNA content prevails and establishes the structural and functional core of the ribosome (Cech, 2000). As mentioned before, the high structural complexity of the ribosomes does not protect rRNA from oxidation (Liu et al., 2012). In a more recent study, we showed that the oxidation of the 50S subunit had more of a negative impact on *in vitro* translation than the oxidation of the 30S subunit (Willi et al., 2018; Figure 1). We observed the same trend with *in vitro* reconstituted 50S and 30S subunits using H_2_O_2_ treated 23S rRNA and 16S rRNA, respectively, in combination with ribosomal proteins isolated from unstressed *Thermus aquaticus*. In both cases, *in vitro* translation of a genuine mRNA was reduced. However, the reduction of translational activity was more severe using oxidized 23S rRNA as compared to 16S rRNA. The 23S rRNA of the 50S subunit harbors the peptidyl transferase center (PTC) of the ribosome. Interestingly, the investigation of the influence of oxidized PTC nucleotides using an atomic mutagenesis approach (Erlacher et al., 2011) showed varied results (Willi et al., 2018). Whereas the oxidation of 23S rRNA residues A2451 and U2585 resulted in a significant loss of translational activity *in vitro*, the oxidation of other active site nucleotides had no effect at all (U2506, A2602, G2447) or resulted in even slightly stimulated protein synthesis as in the case of 5-OH-C2063 (Willi et al., 2018). While these varied results highlight the complexity of oxidative stress damage, the question remains whether these particular nucleotides are actually prone to oxidation *in vivo*. Therefore, Willi *et al.* generated a crude oxidation map of the 23S, 16S, and 5S rRNAs using an 8-oxo-G antibody for immunoprecipitation of oxidized RNA followed by sequencing and alignment to rRNA genes. Fitting to the previous findings, we reported non-random occurrences of oxidation hotspots which were mostly found on the 23S rRNA, including in domain V which harbors the PTC nucleotides (Willi et al., 2018). Remarkably, 16S rRNA regions forming the decoding site and the anti-Shine-Dalgarno sequence of the small subunit appear to remain largely oxidation-damage free *in vivo*, which fits with previous reports that translation initiation was not affected by oxidative stress (Zhong et al., 2015). Efforts for a higher resolution oxidation map of the rRNAs comprising also other rRNA oxidative lesions are currently underway. As mentioned above, oxidation of rRNA inside the ribosome does not necessarily interfere with ribosome functions and oxidation of some residues even resulted in accelerated rates of protein synthesis *in vitro* (Willi et al., 2018). This illustrates that rRNA nucleobase oxidations can potentially be utilized by bacteria to fine-tune ribosome functions during different growth or stress conditions. Indeed, the natural occurrence of the bacterial rRNA modification 5-OH-C2501 gives further credence to this hypothesis. While in the polyextremophile *Deinococcus radiodurans* the modification is found ubiquitously on the bacterium’s ribosomes, it is completely absent in the rather closely related thermophile *Thermus thermophilus* (Havelund et al., 2011). In *E. coli*, the extent of the modification is growth phase dependent. While in early exponential phase only about 30% of ribosomes are modified, the frequency increase in stationary phase to about 70% (Havelund et al., 2011; Kimura et al., 2017). Recent research on the 5-OH-C2501 modification identified RlhA as the modifying enzyme, thereby completing the set of rRNA modifying enzymes in *E. coli* (Kimura et al., 2017). The exact biological role of this cytosine hydroxylation inside the ribosome is however so far unknown.

RNA molecules have a negatively charged phosphate backbone. Magnesium ions (Mg^2+^) are well known for their ability to counteract this negative charge and therefore stabilize the folding of large RNA molecules. Unsurprisingly, there are between 100–1,000 Mg^2+^ ions found on bacterial ribosomes (Zheng et al., 2015) that are essential for the structural integrity and functional activity of the ribosome (Klein et al., 2004). However, it has been shown that these Mg^2+^ ions can be replaced by Fe^2+^ and Mn^2+^ without significant loss of activity, mimicking the conditions of the early anoxic Earth (Athavale et al., 2012; Bray et al., 2018). While it has been shown before that exchange of Mg^2+^ ions in the ribosome can have a detrimental effect on rRNA stability (Winter et al., 1997; Polacek and Barta, 1998), research on the specific role of ribosome bound Fe^2+^ for oxidative lesions generated through localized Fenton reactions is scarce in bacteria. Most of the work has been focused on eukaryotes and human neurodegenerative diseases specifically (Ward et al., 2014; Dusek et al., 2015; Daglas and Adlard, 2018; Smethurst et al., 2020). However, since the core structures and functions of the ribosome are conserved (Melnikov et al., 2012), it is not far-fetched to assume that similar findings can be expected for bacteria. Indeed, our own unpublished data shows a significant increase of 8-oxo-G occurrences at nucleotides that are <2.4 Å from Mg^2+^ binding sites when ribosomes were treated with Fe^2+^ and H_2_O_2_.

Ribosomal proteins have no apparent shielding effect on proximate rRNA against oxidative stress lesions ((Willi et al., 2018), unpublished data). Nevertheless, they are also not spared from oxidative damage. In a global approach to detect redox sensitive cysteines before and after the addition of oxidative stress, Leichert *et al.* identified both small and large ribosomal subunit proteins that had at least one cysteine oxidized (Leichert et al., 2008). Another study found increased carbonylation of ribosomal proteins in viable but non-culturable *E. coli* cells found in a stationary phase culture (Desnues et al., 2003). A specific example of oxidative damage actually inhibiting the biological function of a ribosomal protein was described in two publications in 1978. In the stalk region of the ribosome, protein uL10 (nomenclature according to (Ban et al., 2014)) and multiple copies of the protein bL12 form one of the main interaction sites of the ribosome with translation factors, also known as the GTPase-associated center (Diaconu et al., 2005). The multiple copies of the bL12 protein have been shown to occur in two dimers on the uL10, whereas mutation of the uL10 to a single dimer-binding form showed strongly reduced translational initiation and elongation (Mandava et al., 2012). Oxidation of the three methionine residues found in bL12 to methionine sulfoxide resulted in a loss of dimerization. This leads to inhibited binding of bL12 to uL10 on the ribosome, which in turn leads to a decrease of EF-G dependent translation (see also below) (Caldwell et al., 1978; Koteliansky et al., 1978). Another publication shows an interesting connection between the ribosomal protein uS4, mistranslation, and the resistance to oxidative stress (Fan et al., 2015). The authors found that *E. coli* cells with error-prone ribosomes due to the I199N mutation of uS4 show a significantly lower H_2_O_2_ sensitivity than *E. coli* cells with wildtype ribosomes. Mistranslation of proteins leads to the induction of the general stress response transcription factor RpoS (Battesti et al., 2011). The regulon of RpoS includes the catalase KatE and the peroxidase OsmC, both of which were found to be upregulated in the error-prone *E. coli* strain, explaining the higher tolerance to H_2_O_2_ (Fan et al., 2015). The exact link between mistranslation and induction of RpoS is however not completely clear. The authors speculate that mistranslation of proteins buffers away the protease ClpP, which usually degrades RpoS. This would therefore provide a new pathway to activate the general stress response as a defense mechanism against H_2_O_2_ that is independent of the OxyR regulon, relying on protein mistranslation only. Additionally, the authors find that the deletion of the sRNA DsrA, which is known to positively regulate RpoS translation, results in the loss of the protective effect observed against H_2_O_2_. Mechanistic explanations of this observation remain to be elucidated.

Ribosome biogenesis is a particularly energy intensive process for the bacterial cell. Recycling the whole ribosome after oxidative damage would therefore appear very wasteful. To prevent the incorporation of oxidized free nucleotides in the first place, *E. coli* cells use the protein MutT to hydrolyze both 8-oxo-dGTP and 8-oxo-rGTP to the monophosphate forms, reducing mutagenic errors in DNA and RNA respectively (Taddei et al., 1997). While it is yet unclear whether the damaged rRNA can actually be repaired in cells, there is at least evidence that the polynucleotide phosphorylase (PNPase), which specifically binds oxidized RNA transcripts, also plays a role in proper ribosome assembly (Hayakawa et al., 2001; Cheng and Deutscher, 2003; Wu et al., 2009). In the case of damaged ribosomal proteins, there is evidence that a subset of ribosomal proteins can be exchanged in the fully assembled ribosome, including the above discussed uL10 and bL12 (Pulk et al., 2010).

### Elongation Factors

Elongation factors (EF) are crucial for an efficient translation process. As was discussed above, mostly the elongation step of translation is inhibited by oxidative stress (Figure 1). It would therefore not be surprising, if also the essential elongation factors would be affected by ROS. Indeed, when Tamarit and co-workers analyzed the carbonylation pattern of proteins in *E. coli* after the treatment with H_2_O_2_, EF-G was found to be one of the primary targets (Tamarit et al., 1998). The same was true in *Bacillus subtilis* for both EF-Tu and EF-G (Mostertz and Hecker, 2003). EF-Tu assists the decoding step of elongation. It delivers aminoacylated tRNAs to the free A-site of the ribosome in a ternary complex with GTP. If correct base-pairing of the codon and anti-codon in the decoding center of the 16S rRNA is established, the GTP is hydrolyzed in the GTPase-associated center on the large subunit and EF-Tu is released (Rodnina, 2018). In the cyanobacterium *Synechocystis* sp. PCC 6803 (*Synechocystis*), H_2_O_2_ oxidizes a conserved cysteine of EF-Tu (Yutthanasirikul et al., 2016). The authors further showed that in an *E. coli in vitro* translation system the use of the oxidized EF-Tu resulted in strongly inhibited translation. Similar findings were described for EF-G in both *Synechocystis* and *E. coli* (Kojima et al., 2009; Nagano et al., 2015). EF-G catalyzes the tRNA/mRNA translocation step of elongation, where after a new peptide bond is formed between two amino acids, the ribosome moves along the mRNA to the next codon. Like EF-Tu, EF-G is a so-called G-protein. Its overall structure resembles the structure of the aa-tRNA/EF-Tu complex and it utilizes the hydrolysis of GTP triggered at the ribosomal GTPase-associated center as energy source (Koch et al., 2015; Rodnina, 2018). Upon oxidation with H_2_O_2_, two conserved cysteine residues formed a disulfide bridge which resulted in reduced GTPase activity and suppressed dissociation of EF-G from the ribosome (Kojima et al., 2009; Nagano et al., 2015). Interestingly, mutations of the two target cysteines to serines rendered the EF-G mutant resistant to oxidative stress and did not show significant loss of activity (Kojima et al., 2009). Therefore, the oxidation of these two conserved cysteines is likely a desired and expected mechanism for regulation of translation under adverse condition rather than random oxidative damage.

## Translated Proteins Fulfill Diverse Functions in the Cell

Once translated and properly folded, proteins fulfill a wide array of cellular functions, ranging from structure to catalysis to metabolism and to reproduction. As discussed above in piecemeal, there are several ways how oxidative stress can damage proteins. Carbonylation represents an irreversible and unrepairable damage to proteins. It mostly affects the amino acids proline, arginine, lysine, and threonine and has been used in organisms of all domains of life as an indicator of oxidative damage (Tamarit et al., 1998; Nyström, 2005). Histidine can be oxidized to 2-oxo-histidine as has been shown in the deactivation of PerR through oxidative stress (Lee and Helmann, 2006; Traoré et al., 2009). The amino acids cysteine and methionine both contain an electron-rich sulfur atom in their side chains. Unsurprisingly, they are prone to oxidation through ROS as was seen in the case of OxyR activation (Choi et al., 2001), the loss of editing activity in the ThrRS (Dock-Bregeon et al., 2004), the loss of dimerization of bL12 (Caldwell et al., 1978; Koteliansky et al., 1978), and the loss of both EF-Tu and EF-G activity (Kojima et al., 2009; Nagano et al., 2015; Yutthanasirikul et al., 2016). Cysteine and methionine oxidation is further reviewed in (Ezraty et al., 2017). In the case of singlet oxygen, the impact on free amino acids has been studied extensively *in vitro*. ^1^O_2_ reacts selectively with only a subset of amino acids, namely cysteine, methionine, histidine, tyrosine, and tryptophan, and the interaction of ^1^O_2_ with these residues was found to be strongly solvent dependent (Di Mascio et al., 2019). Studies under more biological relevant conditions, however, confirmed these findings (Michaeli and Feitelson, 1994; Michaeli and Feitelson, 1995; Kim et al., 2008). Importantly, the accessibility of the five vulnerable amino acids in folded proteins (i.e., is the amino acid solvent exposed or buried inside of the protein) can alter the reactivity of a specific residue toward singlet oxygen (McDermott et al., 1991; Michaeli and Feitelson, 1994, Michaeli and Feitelson, 1995; Kim et al., 2008; Jensen et al., 2012; Sjöberg et al., 2016; Di Mascio et al., 2019). A final mechanism of ROS impact on proteins discussed above was the oxidation of iron-sulfur (Fe-S) clusters, as was seen in the case of SoxRS activation (Gaudu et al., 1997). Fe-S clusters are ancient prosthetic groups that could assemble spontaneously on polypeptides of the primordial, anoxic environment found during the first billion years of life on Earth (Imlay, 2006). Today, they are still one of the most ubiquitous cofactors found in nature. Their functions are extremely versatile, ranging from simple iron storage to the regulation of gene expression (Johnson et al., 2005). Both O_2_^−^ and H_2_O_2_ can damage Fe-S clusters by either directly subtracting an electron or by generating reactive HO∙ through a Fenton reaction (Imlay, 2013). Additionally, most often cysteine is the coordinating amino acid of the polypeptide backbone forming the Fe-S cluster (Imlay, 2006). Oxidation of the coordinating cysteine by any generated ROS could lead to the inactivation of the Fe-S protein. Finally, H_2_O_2_ poisons the Isc system, which is responsible for Fe-S cluster synthesis. Therefore, in *E. coli* the OxyR regulon includes the activation of the Suf system, an alternative Fe-S cluster synthesis pathway (Seo et al., 2015).

## Concluding Remarks

Once oxygen levels rose on Earth, bacterial cells had to develop systems to continuously remove endogenously generated ROS to keep oxidative damage to a minimum. Nonetheless, ROS levels inside cells can rise due to a variety of external reasons (Figure 1). Even though bacteria have efficient scavenging and repair systems, ever-emerging research in the field of oxidative stress has shown that all the steps of the central dogma of molecular biology are nonetheless susceptible to oxidative damage. In this review, we focused exclusively on bacterial systems. However, several parts of the central dogma of molecular biology are well conserved among all domains of life thus allowing extrapolation of insights gained in bacteria to other kingdoms. Especially the ribosome, the central hub of translation, has a highly conserved core found everywhere from bacteria to humans (Melnikov et al., 2012) and findings from bacteria can, to an extent and with reasonable caution, be extrapolated to more complex organisms. Nevertheless, there is a plethora of research on oxidative stress directly performed in mammalian systems to be found. Of the outmost interest is the apparent link between oxidative stress and neurodegenerative diseases such as Alzheimer’s and Parkinson’s disease (Barnham et al., 2004). Furthermore, there is an emerging view that these diseases and the associated oxidative stress are influenced by the interplay of the human host and the gut microbiome (Dumitrescu et al., 2018; Luca et al., 2019), highlighting once more the important role of bacteria in human health.
